# Nebulized Pentamidine-Induced Acute Renal Allograft Dysfunction

**DOI:** 10.1155/2013/907593

**Published:** 2013-01-17

**Authors:** Siddhesh Prabhavalkar, Agnes Masengu, Declan O'Rourke, Joanne Shields, Aisling Courtney

**Affiliations:** ^1^Regional Nephrology Unit, Belfast City Hospital, Belfast BT9 7AB, UK; ^2^Department of Histopathology, Belfast City Hospital, Belfast BT9 7AB, UK

## Abstract

Acute kidney injury (AKI) is a recognised complication of intravenous pentamidine therapy. A direct nephrotoxic effect leading to acute tubular necrosis has been postulated. We report a case of severe renal allograft dysfunction due to nebulised pentamidine. The patient presented with repeated episodes of AKI without obvious cause and acute tubular necrosis only on renal histology. Nebulised pentamidine was used monthly as prophylaxis for *Pneumocystis jirovecii* pneumonia, and administration preceded the creatinine rise on each occasion. Graft function stabilised following discontinuation of the drug. This is the first report of nebulized pentamidine-induced reversible nephrotoxicity in a kidney allograft. This diagnosis should be considered in a case of unexplained acute renal allograft dysfunction.

## 1. Background

Drug-induced nephrotoxicity is a potential cause of acute kidney allograft dysfunction in the early posttransplant period [1]. The most common culpable medications are calcineurin inhibitors [2], as other agents associated with nephrotoxicity are generally avoided in this setting. However, there are typically several different new drugs prescribed to patients following kidney transplantation, including prophylactic antimicrobial agents.

Given the higher immunosuppression load in the early post transplant period, there is an increased risk of opportunistic infections like *Pneumocystis jirovecii* pneumonia (PJP). The European Best Practice Guidelines (EBPG) recommend at least four months of PJP prophylaxis postrenal transplantation [3], while the Kidney Disease: Improving Global Outcomes (KDIGO) guidelines suggest 3–6 months [4]. Both guidelines advocate additional prophylaxis during and following the treatment of acute rejection. The recommended treatment of choice is cotrimoxazole. Nebulized pentamidine is an alternative for those patients who are intolerant of cotrimoxazole. 

Acute kidney injury (AKI) has been reported as a complication of intravenous pentamidine therapy. There is a single case report of nebulised pentamidine causing an adverse effect on renal function in native kidneys. We present a case of severe reversible acute kidney allograft dysfunction attributed to the use of nebulized pentamidine therapy.

## 2. Case Report

A 65-year-old woman with end-stage renal disease of unknown aetiology received a renal transplant, from her thirty-two-year-old daughter, after five years of dialysis therapy. Past medical history was significant for hypertension, ischaemic heart disease, and essential thrombocytosis. The kidney was mismatched at B57 and DR7 HLA loci only. There were neither current nor historic HLA donor-specific antibodies detected over five years of regular screening.

The perioperative course was uncomplicated with primary graft function and a creatinine of 92 *μ*mol/L on discharge at day 9 after surgery. The immunosuppression regimen was prednisolone, mycophenolate mofetil, and tacrolimus (trough levels 8–10 *μ*g/L); no induction therapy was used. The patient was allergic to cotrimoxazole and commenced on monthly nebulised pentamidine (300 mg) as prophylaxis for pneumocystis jiroveci. 

Eight weeks after transplantation, the creatinine rose from 80 *μ*mol/L to 140 *μ*mol/L. This followed removal of the ureteric stent but ultrasound imaging of the transplanted kidney showed a well-perfused kidney with no evidence of hydronephrosis. Urine cultures were negative with no clinical features of infection. Biopsy of the graft showed mild acute tubular necrosis with normal appearing glomeruli, mesangium, and interstitium. There was no evidence of rejection or infection and the blood vessels were unremarkable. C4d staining was negative on immunofluorescence. There was a spontaneous improvement in creatinine to 125 *μ*mol/L and the patient was discharged home.

**Figure 1:**
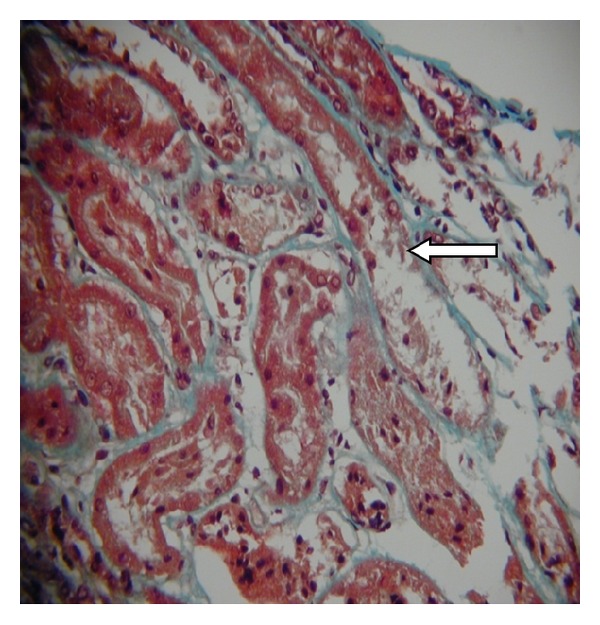
Kidney allograft biopsy: light microscopy showing acute tubular necrosis (arrow marked with magnification × 200).

Ten days later the creatinine had risen again. A further biopsy demonstrated moderate acute tubular necrosis only (Figure 1). There were no donor-specific antibodies. The ultrasound scan appearances were unchanged, but given that the initial deterioration in function occurred after stent removal and the continued decline, a nephrostomy tube was placed. However, there was free flow of contrast medium from the renal pelvis into the bladder and no improvement in function. A renal angiogram showed no evidence of iliac or anastomotic stenosis. The creatinine rose to 329 *μ*mol/L but then spontaneously improved. The patient was discharged home with a creatinine of 234 *μ*mol/L. 

Twelve days later the patient's creatinine was 556 *μ*mol/L. A third renal biopsy was carried out which again demonstrated moderate acute tubular necrosis with no evidence of any additional pathological process. Ultrasound scan appearances were unchanged, and a repeat cross-match with the donor was negative. The tacrolimus dose was reduced with subsequent trough levels of 2–8 *μ*g/L. Over the next two weeks, renal function again improved, and creatinine at discharge was 145 *μ*mol/L. There was one further episode of deterioration in function, with a peak creatinine of 215 *μ*mol/L, which settled spontaneously.  

**Figure 2:**
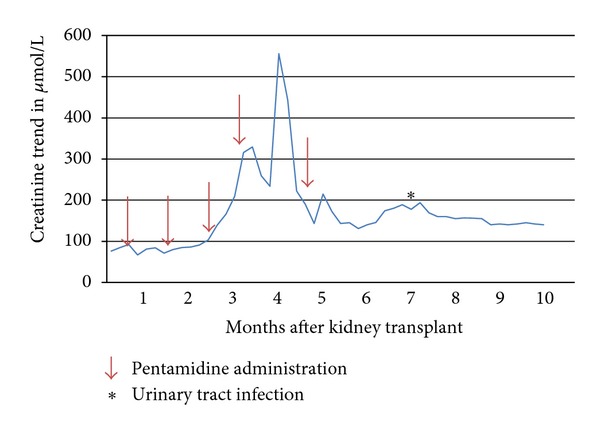
Trend of creatinine levels (*μ*mol/L) with arrows indicating pentamidine doses.

Thus there were recurrent episodes of AKI, without explanation, with acute tubular necrosis only on renal biopsy. The timing of graft dysfunction in relation to pentamidine administration is shown in Figure 2. Hospitalisation interrupted the monthly administration of pentamidine but the patient received the prescribed dose after discharge, and there was a subsequent deterioration again in renal function. Discontinuation of pentamidine has resulted in a sustained improvement in renal function. 

## 3. Discussion

There have been a number of reports of PJP outbreaks in renal transplant recipients worldwide in recent years [5]. Prophylactic therapy has been increasingly used in this population, consistent with the recommendations in the EBPG and KDIGO guidelines. Cotrimoxazole (a combination of sulphamethoxazole plus trimethoprim) is the drug of choice but has a well-recognised list of side effects and can be poorly tolerated. The alternative options for prophylaxis are nebulised pentamidine or dapsone. The former is attractive because of infrequency of administration (four weekly) and typically the adverse effects associated with parenteral administration are not experienced. In our institution, we use it preferentially to dapsone when cotrimoxazole is not tolerated.

Reversible AKI is a relatively common complication of intravenous pentamidine therapy, occurring in over 25% of cases in some series [6–8]. Most of these case reports of pentamidine-induced nephrotoxicity concerned Human Immunodeficiency Virus (HIV) infected patients receiving treatment doses. The mechanism by which pentamidine produces AKI is unclear, but a direct nephrotoxic effect leading to acute tubular necrosis has been postulated. The likelihood of developing AKI is increased in those patients who are volume depleted or who are exposed to other tubular toxins that could act synergistically with pentamidine. Examples include aminoglycoside antibiotics, amphotericin B, and foscarnet [9]. 

There is a single case report in 1989 reporting nephrotoxicity in native kidneys with nebulized pentamidine [10]. There have been no reports of an adverse effect of a nebulized prophylactic dose of pentamidine on renal transplant function. 

A multitude of factors can influence graft function in the early post transplant period; however, none of the potential causes of acute kidney injury were culpable in this case. The absence of any other plausible explanation, the finding of acute tubular necrosis on biopsy, the timing of graft dysfunction in relation to drug administration, and the stability of creatinine after discontinuation of pentamidine supports the hypothesis that this was the aetiology of the acute kidney injury. The fact that this drug was not listed in the prescribed medicines during the in-patient stay contributed to the delay in considering this as a potential cause of graft dysfunction. This is the first reported case in the literature of nebulized pentamidine-induced reversible nephrotoxicity in a kidney allograft recipient and highlights the importance of considering it as a potential cause of unexplained graft dysfunction.
